# Identifying strokes in Nigerian children with sickle cell disease as part of clinical trials: training curriculum for healthcare professionals in low-income settings

**DOI:** 10.3389/fstro.2024.1444718

**Published:** 2025-01-06

**Authors:** Djamila L. Ghafuri, Halima Bello-Manga, Fenella J. Kirkham, Mariana Ciobanu, Edwin Trevathan, Mark Rodeghier, Michael R. DeBaun, Lori C. Jordan

**Affiliations:** ^1^Department of Pediatrics, Division of Pediatric Neurology, Vanderbilt University Medical Center, Nashville, TN, United States; ^2^Department of Hematology and Blood Transfusion, Barau Dikko Teaching Hospital/Kaduna State University, Kaduna, Nigeria; ^3^Developmental Neurosciences, University College London Great Ormond Street Institute of Child Health, London, United Kingdom; ^4^Vanderbilt Institute for Global Health, Vanderbilt University Medical Center, Nashville, TN, United States; ^5^Rodeghier Consultants, Chicago, IL, United States; ^6^Department of Pediatrics, Vanderbilt-Meharry Sickle Cell Center for Excellence, Nashville, TN, United States

**Keywords:** stroke prevention, children, sickle cell anemia, low-resource setting, Nigeria, sub-Saharan Africa, curriculum

## Abstract

**Introduction:**

Nigeria has the highest proportion of children with sickle cell anemia (SCA) globally; without transcranial Doppler screening and ongoing treatment (regular blood transfusions or hydroxyurea therapy), 10% will have a stroke in childhood. In low-resource settings, training to recognize and prevent strokes in children with SCA is vital. A sustainable *Sickle Cell Disease Stroke Prevention Teams* program was established, as part of clinical trials, to address the need for stroke care in northern Nigeria. We describe our health professional stroke training curriculum and specific application to detect strokes in clinical trials in low-resource settings.

**Methods:**

Children aged 5–12 and 2–16 years with SCA in northern Nigeria were enrolled in the SPRING and SPRINT primary and secondary stroke prevention trials, respectively. The primary outcome measure in both trials was a clinical stroke based on the World Health Organization definition. Non-neurologist physicians were trained in-person and via video lectures regarding stroke recognition, performing neurological examinations using the adapted Pediatric NIH Stroke Scale, and acute stroke care. Central stroke adjudicators, two pediatric neurologists, reviewed the case report forms and recorded videos of the neurological examinations.

**Results:**

Six physicians completed the curriculum at three sites and were certified to detect strokes. Of 20 children with suspected stroke, 8 and 11 children had acute initial or acute recurrent strokes confirmed in the SPRING (*N* = 220) and SPRINT (*N* = 101) trials, respectively. The concordance rate between local stroke diagnoses and the central stroke adjudication process was 95% (19 of 20). One child presented with non-specific symptoms and hypertonia and was mislabeled locally as an acute stroke.

**Discussion:**

A curriculum to train healthcare providers in pediatric acute stroke recognition and care in a low-resource setting is feasible and sustainable. We successfully identified strategies for task shifting from a single pediatric neurologist in the region to multiple non-neurologist physicians.

## 1 Introduction

Nigeria has the highest proportion of children with sickle cell anemia (SCA) globally; an estimated 150,000 infants with SCA are born annually (WHO, 2006). Stroke occurs in about 10% of children with SCA without primary stroke prevention; without secondary stroke prevention, approximately 67% of children with SCA and a first stroke will have a recurrent stroke within 3 years (Powars et al., 1978). In children with SCA, transcranial Doppler (TCD) velocities that are abnormal (>200 cm/sec) and conditional (170–199 cm/sec) in internal carotid and middle cerebral arteries predict a 40% and 7% risk of stroke over the subsequent 40 months (Adams et al., 1997). In high-income countries, primary stroke prevention for children with SCA consisting of TCD screening coupled with regular blood transfusion therapy for those with abnormal TCD velocities is associated with a 92% reduction in stroke incidence (Adams et al., 1998) rate after 1 year of transfusion, transitioning to the maximum-tolerated dose of hydroxyurea appears to be non-inferior in children without intracranial stenosis (Ware et al., 2016).

Gaps exist in acute stroke diagnosis and management in low and middle-income countries (LMICs) with less robust neuroimaging and emergency medical systems. Neuroimaging is limited or absent in many low- and middle-income countries, where the diagnosis of stroke is often dependent upon clinical criteria.

In a low-resource setting, major barriers exist to primary and secondary stroke prevention in children with SCA: (1) availability, cost, and safety of regular blood transfusions compelling most families to decline this long-term intervention (Lagunju et al., 2013) and (2) few healthcare professionals have been taught how to detect and manage acute strokes in this high-risk population. Our team successfully built and maintained pediatric SCA primary stroke prevention programs, including radiologists and non-radiologists trained and certified to perform TCD, scalable e-prescriptions, and offering hydroxyurea therapy for free for children with SCA at high risk for stroke in three hospitals in northern Nigeria (Ghafuri et al., 2021). These capacity-building and quality improvement efforts were components of three stroke prevention trials in SCA in northern Nigeria (Stroke Prevention in Nigeria [SPIN] trial, NCT01801423), Primary Prevention of Stroke in Children with Sickle Cell Disease in Nigeria, Stroke Prevention in Nigeria [SPRING] trial, (NCT02560935) and SPRINT, a trial focused on secondary stroke prevention in children with SCA, (NCT02675790) all utilized oral hydroxyurea for stroke prevention. Caregivers of children enrolled in the trials with possible stroke were advised to both notify study staff and to bring children to the hospital to be examined by physicians trained to perform a standardized neurological exam, the Pediatric National Institutes of Health Stroke Scale (PedNIHSS) (Ichord et al., 2011). We describe our curriculum for healthcare providers with objectives focused on pediatric acute stroke recognition and pediatric stroke acute care in a low-resource setting, and this curriculum's application in these trials where a clinical stroke was the primary outcome.

## 2 Materials and methods

### 2.1 Study design and participants in three stroke prevention clinical trials

The curriculum for identifying strokes in children without neuroimaging was developed and integrated into training for study personnel training for the clinical trials. These three clinical trials are summarized in this section; all had clinical stroke as a trial endpoint.

Study participants included children with SCA ages 5 to 12 enrolled in the SPIN and SPRING trials and 1 to 16 years of age for the SPRINT trial in northern Nigeria. The trial participants were from Aminu Kano Teaching Hospital (AKTH), Murtala Mohammed Specialist Hospital (MMSH) in Kano, Nigeria, and Barau Dikko Teaching Hospital/Kaduna State University (BDTH), Kaduna, Nigeria. This catchment area has at least 40,000 children with SCA (Abdullahi et al., 2022). The studies received ethical approval from the Institutional Review Boards of Vanderbilt University Medical Center, Nashville, United States, and the three sites in Nigeria.

SPIN, a feasibility trial, determined the acceptability of hydroxyurea therapy for primary stroke prevention in children with abnormal transcranial Doppler (TCD) measurements (April 2013–October 2019) (Galadanci et al., 2020). Twenty-nine children with SCA and abnormal non-imaging TCD measurements (≥200 cm/s) received moderate fixed-dose hydroxyurea therapy (~20 mg/kg/day); 206 participants with TCD velocities < 200 cm/sec were enrolled as controls. One stroke occurred in a participant with a conditional TCD (170–199 cm/sec) in the control group.

The SPRING trial was a double-blind, parallel-group, randomized controlled phase 3 trial of 220 children (5–12 years) with SCA and abnormal TCD velocities conducted at three teaching hospitals in Nigeria (August 2016–March 2020). The trial compared moderate-dose to low-dose hydroxyurea for primary stroke prevention (Abdullahi et al., 2022).

The SPRINT trial was a single-center, double-blind, randomized controlled trial for secondary stroke prevention that included children with SCA ages between 1 to 16 years of age with recent overt strokes (*N* = 101) randomized within 30 days, to receive either low- or moderate-dose hydroxyurea assessing stroke recurrence rate (January 2017–March 2020). The Afolabi Stroke Registry for Children and Young Adults with SCD in Northern Nigeria (NCT04800809) was created to document the natural history of SCD in children and young adults with SCD living in northern Nigeria. The trial methodology and results are published (Abdullahi et al., 2023).

### 2.2 Standardized neurological evaluation and documentation for clinical trial participants

Participants in all three trials completed monthly research visits, which included a stroke-free questionnaire and a standardized neurological evaluation (the PedNIHSS; Supplementary material 1) (Ichord et al., 2011). If there was a concern for study participants having an initial stroke for SPIN or SPRING or a recurrent stroke for SPRINT as judged by the Nigerian treating or trial physicians, a physician trained in stroke recognition performed a complete neurological examination and video-recorded the PedNIHSS to document deficits with an iPad. These videos were uploaded to the study REDCap database (Harris et al., 2009, 2019). The Neurology Committee reviewed the neurological examination video and case report forms of all suspected strokes blinded to group assignment. The neurology committee included two pediatric neurologists with pediatric stroke expertise (LCJ and MC). The neurology committee made the final decision to categorize a neurological event as a stroke.

### 2.3 Acute pediatric stroke care curriculum in northern Nigeria

#### 2.3.1 The world health organization's definition of stroke was used

The primary endpoint in the primary stroke prevention (SPIN/SPRING) and secondary stroke prevention (SPRINT) trials was an initial or recurrent clinical stroke, respectively, based on the WHO definition. The traditional definition of stroke endorsed by the WHO requires clinical symptoms for >24 h and has been used for decades (Aho et al., 1980). Computed tomography (CT) is relatively expensive for families and may not detect stroke within 24 h of onset. In this region, head CT was only available at an off-site private facility, thus CT was not an option for acute stroke diagnosis. An MRI-based definition of stroke was challenging due to the limited number of MRI scanners and radiologists in low–middle–income settings. For these reasons, none of the children in these trials had neuroimaging investigations.

#### 2.3.2 Caregiver education on early recognition of stroke symptoms to ensure timely evaluation and management

Widespread education of families and patients about the early recognition of stroke symptoms and signs is crucial in acute pediatric stroke care. Evidence-based tools (“FAST = Face Arm Speech Time”) (Yock-Corrales et al., 2011) were used to educate caregivers on recognizing stroke signs and symptoms to ensure timely transport of children with SCA and possible acute stroke to the hospital for further evaluation and management, including transfusion and supportive care. Signs and symptoms included any arm or leg weakness, facial drooping, speech difficulty, unsteady gait, visual deficits, focal sensory symptoms alone or in combination with other symptoms such as seizure and severe headache. At AKTH and BDTH, nurses lead weekly educational sessions for caregivers about SCD and stroke in the hospital waiting area. On average, approximately 125 caregivers attend these sessions lasting 15 min before their children are seen in the SCD clinic. Feedback from these sessions from caregivers was positive, but we did not conduct pre- and post-education assessments.

#### 2.3.3 Modified pediatric NIH stroke scale implementation as a basic screening neurological examination and training of study personnel in performing neurological examination

The curriculum for healthcare providers emphasized early recognition and acute stroke care in the pediatric population in northern Nigeria. In prior studies, neurologic examination had 58% sensitivity and 92% specificity for cerebral infarction in children with SCA (DeBaun et al., 2014). The Pediatric Acute Stroke Care curriculum included training on the PedNIHSS assessment (Aho et al., 1980), stroke symptoms, types of strokes, pathophysiology, and treatment options; online training modules were utilized; five video lectures (Table 1) and a video recording of a pediatric neurological examination were completed (Supplementary material 1 PedNIHSS Pocket Guide for Nigeria Modified to Identify Acute and Chronic Stroke; Supplementary material 2 Video-lectures).

**Table 1:** Lecture topics: pediatric stroke detection and management in children with sickle cell disease in low resource settings^*^.

Stroke in Pediatric Sickle Cell Disease Overview
Pediatric Neurology History and Examination
Pediatric Neurology History and Exam Suggesting Stroke
Pediatric National Institutes of Health (NIH) Stroke Scale Part 1
Pediatric NIH Stroke Scale Part 2
Supportive Care after Ped Stroke in low resource settings

^*^Links to the lecture videos are in the Supplementary material.

A group of Nigerian research physicians from the three hospitals were trained and certified to perform a modified version of the PedNIHSS by two pediatric neurologists with pediatric stroke expertise (LCJ and FJK).

The PedNIHSS was modified for objects appropriate for Nigerian children (e.g., naming a picture of a soccer ball rather than a baseball, pants instead of a buttoned shirt, and a fish instead of a skateboard) (see Supplementary material 1) (Ichord et al., 2011). We were advised by local pediatric physicians from Northern Nigeria that reading was not a consistent skill in a region with low literacy rates and would not reliably distinguish children with strokes. Reading words and sentences was removed. To assess speech fluency and word finding, instead of the “cookie thief” and “washing dishes with the sink overflowing” pictures where the child is asked to describe what they see, Nigerian children were asked to describe 2 more culturally relevant picture scenes: a child chasing a goat and an adult reading to a group of children. Again, these scenes were selected with local physician advice. The standard PedNIHSS includes adaptations for very young children 4 months to 2 years and toddlers 2 years or more with limited verbal skills (point to mommy). The adaptations that we made such as picture naming only apply to older, verbal children. A printed laminated pocket-sized book containing instructions for the PedNIHSS for Nigerian children was provided to each trained study physician (see Supplementary material 1) (Ichord et al., 2011) The PedNIHSS was used with the “10 questions” screening questionnaire for developmental delays as well as neurological issues (Mung'ala-Odera et al., 2004), and caregiver history to assess for acute stroke or history of stroke. Several additional assessments including muscle bulk, tone, gait, and arm swing, are not part of the PedNIHSS, but were included as part of the neurological assessment, to help identify whether a stroke was acute or chronic. Children presenting with acute strokes typically have low muscle tone along with focal weakness. In contrast, children with subacute or chronic stroke often have increased muscle tone (spasticity) in the affected limbs. As part of the pediatric stroke curriculum, we taught non-neurologist physicians to assess for signs of prior, chronic stroke not previously recognized. We asked that they consider the following questions when evaluating a child, as each could be subtle sign of unrecognized prior stroke: (1) Is there atrophy on one side of the body? If yes, there is concern for chronic stroke. Look at forearm, hand size, and lower leg/calf muscle. Are these areas of the body smaller? (2) Is there increased muscle tone/tightness on one side of the body? (3) Gait: Does the child drag one leg, or does one leg turn out slightly and not lift as high off the ground when the child walks? (4) Does one arm swing less than the other arm when walking? Recall that a baseline PedNIHSS was conducted prior to study enrolment. If any of these findings were present, this child was not to be enrolled.

#### 2.3.4 Acute and subacute stroke management

**Figure 1:**
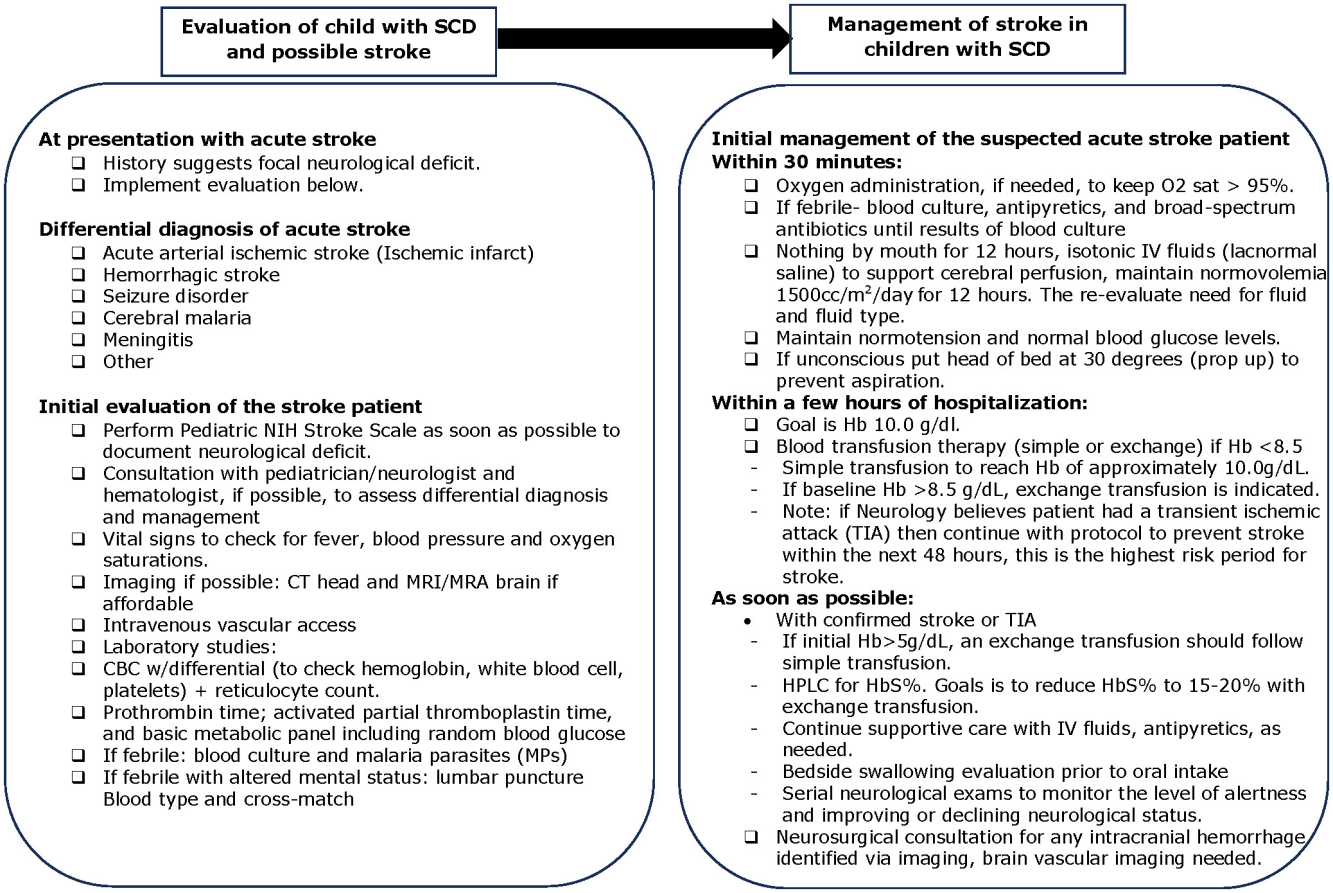
Acute and subacute management of overt stroke in peds SCD in Kano and Kaduna, Nigeria.

In low-resource settings such as northern Nigeria, the acute and subacute management of stroke in children with SCA necessitates a streamlined approach to ensure timely and effective interventions, Figure 1. Initial management of patients with suspected stroke within 30 min of clinical presentation includes essential measures such as maintaining oxygen saturation above 95%, administering oxygen when needed, testing for malaria, obtaining blood cultures, and initiating antibiotics if febrile, performing a lumbar puncture if febrile with altered mental status, giving isotonic IV fluids to support and maintain cerebral perfusion, usually for 24 h. Normotension and normal blood glucose levels were recommended (Grelli et al., 2016), and unconscious patients were positioned with the head of the bed elevated at 30 degrees to prevent aspiration. Within the first few hours of hospitalization, the goal was to achieve a hemoglobin level of 10.0 g/dL. Depending on baseline hemoglobin levels, either simple (if baseline hemoglobin < 8.5 g/dL) or manual exchange transfusion (if baseline hemoglobin 8.5 g/dL and above) was recommended to reach a target hemoglobin level of 10.0 g/dL and to decrease the possibility of hyperviscosity syndrome by reducing the percentage of hemoglobin S (DeBaun et al., 2020).

If a child was diagnosed with a transient ischemic attack (TIA) with focal neurological symptoms resolving in < 24 h, they were managed according to the protocol above to prevent stroke within the next 48 h, which is the highest risk period for stroke. Reducing hemoglobin S levels to 15%−20% via exchange transfusion, when possible, is recommended but this was generally not possible for TIA in this low-resource setting (Faye et al., 2017). A bedside swallow evaluation was recommended before oral intake. Close clinical monitoring and serial neurological examinations were crucial to monitoring patient alertness and neurological status. Neuroimaging was not available during the clinical trials discussed, however, if a patient with SCA had been identified with intracranial hemorrhage via imaging, further brain vascular imaging, and neurosurgical consultation would be recommended, given the increased risk of cerebral aneurysm in SCA (Nabavizadeh et al., 2016). For post-stroke rehabilitation, families were guided to perform simple exercises to reduce motor spasticity and contractures while improving the range of motion.

## 3 Results

### 3.1 Clinical trial results pertaining to the curriculum

A total of 20 children enrolled in the SPIN, SPRING and SPRINT studies presented to the hospital with stroke-like symptoms. One child enrolled in the SPIN trial was evaluated for a new acute stroke but instead was recognized to have subtle deficits from a remote stroke (increased muscle tone and spasticity) but no evidence of a new stroke and was subsequently excluded from the trial analysis. Eight children had acute primary strokes in the SPRING trial, and 11 had acute-on-chronic, i.e., recurrent, strokes in SPRINT. Seven of the 19 children (36.8%) were male, with a median age of 6.2 years (interquartile range, IQR, 5.5–8.3). The median interval between strokes after enrolment in the primary and secondary stroke prevention trials was 12.3 months (IQR, 9.0–13.5) and 10.3 months (IQR, 4.6–14.8), respectively. Table 2 shows the demographics and presenting symptoms of the 19 children with acute stroke.

**Table 2:** Clinical features of stroke and PedNIHSS in trials of hydroxyurea for stroke prevention in sickle cell disease in Nigeria, SPRING (primary prevention) and SPRINT (secondary prevention).

**Patient number and group**	**Age (yrs) at study entry**	**Sex**	**Time from beginning hydroxyurea to stroke (mo)**	**Time from symptom onset to presentation for care** **(days)**	**Clinical features of stroke and pediatric NIHSS**
1 – Primary stroke prevention	9.1	Female	8.2	Same day, admitted from clinic	Slurred speech, right hemiparesis, face, and arm more than leg; PedNIHSS = 5.
2 – Primary stroke prevention	10.0	Male	12.2	4 days after event	Left hemiparesis, leg more than arm; PedNIHSS = 3.
3 – Primary stroke prevention	7.4	Female	10.0	Same day, admitted from clinic	Right face, arm, and leg weakness; PedNIHSS = 4.
4 – Primary stroke prevention	6.2	Female	0.3	Same day	Right hemiparesis, arm more than leg; PedNIHSS = 3.
5 – Primary stroke prevention	8.3	Female	10.4	Same day	Left hemiparesis, face and arm more than leg, left visual field cut; PedNIHSS = 5.
6 – Primary stroke prevention	12.4	Male	8.0	Same day	Left hemiparesis, arm more than leg; PedNIHSS = 2.
7 – Primary stroke prevention	5.1	Male	14.8	Next day	Left-sided seizure, with persistent hemiparesis, face, and arm more than leg; PedNIHSS = 7.
8 – Primary stroke prevention	6.5	Male	30.7	Next day	Seizure, global aphasia, bilateral weakness, right more than left, bilateral strokes in setting of Escherichia coli sepsis; PedNIHSS = 26.
1 – Secondary stroke prevention	5.6	Female	9.5	Next day	At baseline had mild left-sided weakness and significant right hemiparesis which improved over the course of the study. Then presented with acute worsening of prior mild left-sided weakness, PedNIHSS 11. Adjudicated as new stroke.
2 – Secondary stroke prevention	5.5	Female	3.4	Same day	Seizure and new left hemiparesis. Prior deficits were mild right hemiparesis. Ped NIHSS increased from 3 to 28.
3 – Secondary stroke prevention	6.0	Female	19.0	Same day	Sudden onset of right hemiparesis and aphasia. Prior deficit was mild left hemiparesis. PedNIHSS increased from 3 to 14.
4 – Secondary stroke prevention	4.0	Female	17.8	4 days after event	Sudden worsening of baseline left hemiparesis, now unable to walk without handheld. PedNIHSS increased from 4 to 6.
5 – Secondary stroke prevention	5.4	Female	8.8	Next day, admitted from clinic	Seizure followed by persistent worsening of left hemiparesis. PedNIHSS increased from 5 to 8.
6 – Secondary stroke prevention	6.6	Male	4.5	Same day	Seizure followed by persistent worsening of left hemiparesis. PedNIHSS increased from 3 to 8. No longer has any use of left hand.
7 – Secondary stroke prevention	3.8	Female	7.4	Same day	Acute onset right hemiparesis, face and arm with dysarthria. PedNIHSS was 3 at baseline was 3 and at the time of new event was 6.
8 – Secondary stroke prevention	11.2	Female	3.0	Same day	Presented with focal seizure and worsened right hemiparesis, PedNIHSS increased from 3 to 5. New deficits persisted.
9 – Secondary stroke prevention	6.1	Male	4.8	7 days later	During a pain crisis, sudden onset of new left-sided hemiparesis in a child with prior right hemiparesis. PedNIHSS increased from 10 to 15.
10 – Secondary stroke prevention	7.3	Male	23.6	Same day	Fever with seizure and new left hemiparesis. Prior deficits were right hemiparesis. PedNIHSS increased from 7 to 24.
11 – Secondary stroke prevention	5.8	Female	11.9	Same day	Sudden, profound aphasia and weakness of all 4 extremities. PedNIHSS was 6 at study entry due to right-sided weakness. PedNIHSS worsened to 19 for right and left-sided weakness, consistent with bilateral infarcts.

While the exact presentation time was not recorded, the date of symptom onset and presentation for care were documented. Most children, 14/20 (70%), presented on the same day as stroke onset and were evaluated in the emergency department or the SCD clinic. A few children, 6/20 (30%), did not present for care for care until the following day (3 children) or several days (3 children). Delays in presentation for these 6 children seemed to be due to: the co-occurrence of stroke symptoms with illness (2 children) or pain episodes (1 child), the cost of transportation/distance to the hospital (2 children), and or reasons for the delay unclear (1 child). Children received supportive care along with simple transfusion or manual exchange transfusion, or both, at the time of acute stroke per the acute care protocol in Figure 1 (DeBaun et al., 2020). Caregivers in all three trials were offered regular blood transfusions as the first option for primary and secondary stroke prevention; however, none of the caregivers agreed to initiate regular blood transfusion therapy. As standard care, if a child had a stroke during the trial, hydroxyurea therapy was offered and initiated for secondary stroke prevention at a higher dose than they were receiving in the clinical trial setting, so if a child was receiving 10 mg/kg/day of hydroxyurea, the dose was increased to 20 mg/kg/day. If a child was receiving 20 mg/kg/day when a stroke occurred, then the hydroxyurea dose was increased to the maximum tolerated dose.

### 3.2 Physicians trained and concordance with central stroke adjudication

A total of six non-neurologist physicians, three recent medical school graduates (general medical officers), and three pediatric physicians at three participating sites in northern Nigeria successfully completed the curriculum, defined as three observed neurological exams graded as appropriate by training physicians. The concordance rate between local stroke diagnoses and the central stroke adjudication process was 95% (19 of 20). Early in the feasibility trial, one participant initially thought to have an acute stroke by the local team was found to have increased tone and atrophy on one side of their body, indicating an old stroke. Based on this finding, the PedNIHSS in Nigeria (Supplementary material 1) was adjusted with additional instructions on assessing children for chronic vs. acute strokes. The curriculum was iterative; modifications and improvements were made over time.

### 3.3 Unforeseen barriers for stroke prevention teams

Several barriers were identified based on feedback from the locally trained research personnel and on viewing and reviewing the recorded neurological examinations. Perhaps the biggest challenge was the recognition of strokes by non-research-trained nurses, primary care providers, and emergency department physicians. Before the start of the Stroke Prevention Teams, knowledge regarding stroke as a frequent complication of SCA in children was low, and there was no concerted effort to identify strokes in children with SCA. Further, prior to the clinical trials, even after a stroke was identified, there was no established standard care approach for the management of acute stroke and secondary stroke prevention in this region. Thus, there was limited clinical incentive to identify a stroke even if one was present because of the restricted use of regular blood transfusion for secondary stroke prevention. To address these gaps, active surveillance in the Emergency Department was initiated to identify children with strokes; both physicians and nurses received training and education from trial physicians regarding an acute stroke protocol for children with SCA (Figure 1).

## 4 Discussion

Strokes in children with SCA are common in sub-Saharan Africa (Marks et al., 2018). Unfortunately, to our knowledge, limited teaching in textbooks or online exists regarding strategies to prevent, detect, and treat acute strokes in children with SCA, particularly in Africa, where over 75% of all children with SCA are born (Esoh et al., 2021). As part of these stroke prevention studies in children with SCA in Northern Nigeria, we developed a pediatric stroke care curriculum to educate local healthcare providers to improve early recognition and management of stroke in SCA, including acute treatment, supportive care, rehabilitation, and long-term stroke prevention strategies, in a low-resource setting. Our formal stroke education curriculum consisted of in-person and video-based training materials (Supplementary material 2), including the adapted PedNIHSS pocketbook (Supplementary material 1) and weekly meetings with the research team to discuss any suspected strokes or clinical care management concerns.

Several key challenges encountered during this study period were identified. The shortage of pediatric neurologists in this region poses a barrier to the accurate assessment of children with possible strokes using the PedNIHSS. Only one of our trial co-investigator physicians was a trained neurologist. He was unable to personally evaluate children with acute strokes in the three trials due to clinical workload. Due to the shortage of pediatric neurologists in the region, we anticipated the need for additional training of non-neurologists to identify acute strokes in children. Based on our experiences with the SPIN, SPRING, and SPRINT trials, we see the critical importance of shifting tasks from pediatric neurologists to primary care providers and nurses. Our curriculum for the stroke prevention teams taught non-neurologists the necessary skills and techniques to conduct thorough neurological assessments in pediatric patients, ensuring more accurate and timely identification of stroke, particularly in young children with SCA.

Though our small sample size limited generalizability, we believe our study is valuable as a first step in training non-neurologist healthcare providers to detect stroke in children and care for children after an acute stroke. Unfortunately, we did not do formal pre- and post-curriculum assessments. We developed lectures and teaching with direct feedback from senior trial pediatric neurologists, as we would train any physician who had graduated medical school but was embarking on additional clinical training. We are not able to go back and conduct pre- and post-curriculum assessments. Our general observation was that medical officers, physicians who were recent medical school graduates, were understandably less experienced with children than pediatricians. They often needed more guidance in methods to encourage children to cooperate with the neurological exam. Feedback was given during PedNIHSS training and after viewing the video recordings.

Task-shifting from the limited number of pediatricians in the SCA clinic to nurses providing direct patient care to identify acute strokes in children was felt necessary for long-term sustainability in Northern Nigeria. To facilitate this task-shifting, both written materials and a formal curriculum were developed for a self-taught curriculum offered to all nurses, specifically at Murtala Muhammed Specialist Hospital, where nurses provide sickle cell disease medical care to over 20,000 children with SCA. Work in this area is ongoing.

In low-income countries such as Nigeria, limited access to healthcare facilities, especially those equipped with neurodiagnostic tools and specialized pediatric stroke care, are primary barriers. Additionally, a lack of available pediatric neurologists can lead to delayed or missed stroke diagnoses in children. Low awareness among caregivers and communities about the association between SCA and stroke and the signs and symptoms of stroke may further contribute to delays in seeking medical care. Other prominent issues identified were delays in patient presentation for medical care, due to the distance to the study hospital, financial constraints on traveling to the hospital, and paying out of pocket for the necessary medical care. This delay in seeking medical attention could potentially hinder the effectiveness of acute stroke interventions, although these were also very limited in this setting.

Challenges in stroke identification included difficulty in accurately scoring the PedNIHSS via video review due to the inability to discern whether a child was unwilling to participate due to fear or was unable to follow commands due to receptive aphasia. Furthermore, local modesty concerns led to several children being assessed while fully clothed (for example, wearing a long skirt), potentially affecting the accuracy of neurologic assessments. However, prompt identification of this issue and subsequent modifications were implemented to address these challenges and improve the neurological assessment by the acute stroke teams, including removing obscuring clothing in a manner that preserved modesty (changing skirts to pants) and providing exam strategies that helped assess whether commands could be followed (engaging caregivers to help examine children, encouraging the participant during the neurological exam).

The limited availability of post-stroke physical rehabilitation within this region presents a significant challenge to the comprehensive management of pediatric stroke. While these interventions may offer some benefits in terms of symptom management and functional improvement, the absence of formalized rehabilitation programs likely impacts the overall recovery trajectory after stroke. Long-term outcomes were not a component of the studies that were conducted. Our initial focus was to educate non-neurologists on how to diagnose and manage an acute stroke.

A curriculum to train healthcare providers in pediatric acute stroke recognition and care in a low-resource setting is feasible and sustainable. We successfully identified strategies for task shifting from a single pediatric neurologist in the region to multiple non-neurologist physicians.

## Data Availability

The original contributions presented in the study are included in the article/Supplementary material, further inquiries can be directed to the corresponding author.
