# Supplementary material for: Identifying strokes in Nigerian children with sickle cell disease as part of clinical trials: training curriculum for healthcare professionals in low-income settings
Source: Front Stroke. 2025 Jan 6;3:1444718. doi: 10.3389/fstro.2024.1444718 (PMC12802787; doi:10.3389/fstro.2024.1444718)
Supplement: Supplementary file 1 [file Data_Sheet_1.PDF]

Pediatric NIH Stroke Scale in Nigeria

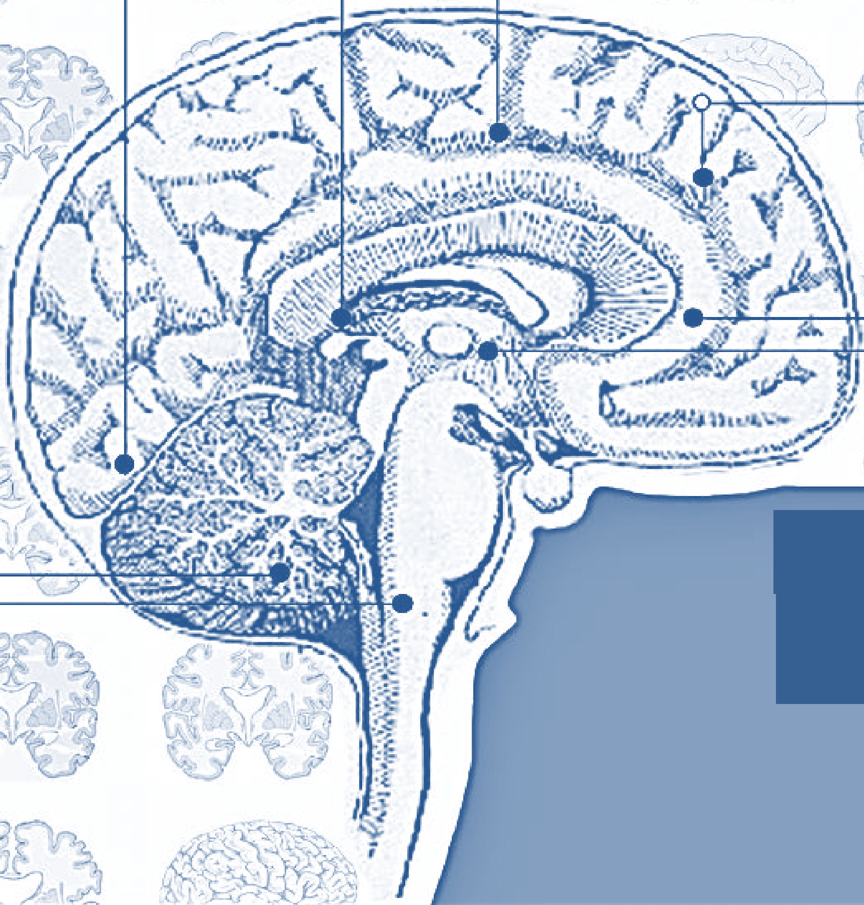

Updated for acute and chronic stroke

www.pednihss.com

Pocket Guide

Instructions

<u>Acute/subacute strokes occurring in the last few days:</u>

- Onset of symptoms should be sudden.
- A child with a new stroke has low muscle tone on the weaker side of the body.
- A child with a new stroke has no atrophy.

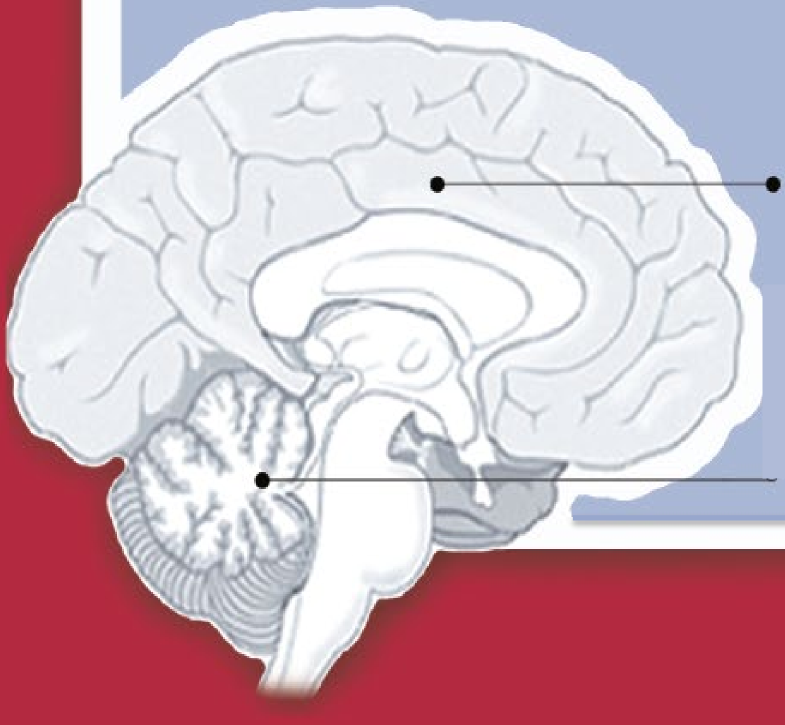

Instructions

*Overview: **Members of the healthcare team should assess for signs of prior/chronic stroke not previously recorded**: Look for asymmetry, atrophy, high muscle tone, and gait abnormalities.

1) Is there atrophy on one side of the body? If yes, there is concern for chronic stroke. Look at forearm, hand size, and lower leg/calf muscle. Are they smaller?
2) Is there increased muscle tone/tightness on one side of the body? (chronically)
3) Gait: Does the patient drag one leg, or does one leg turn out slightly and not lift as high off the ground when the patient walks?
4) Does one arm swing less than the other arm when walking?

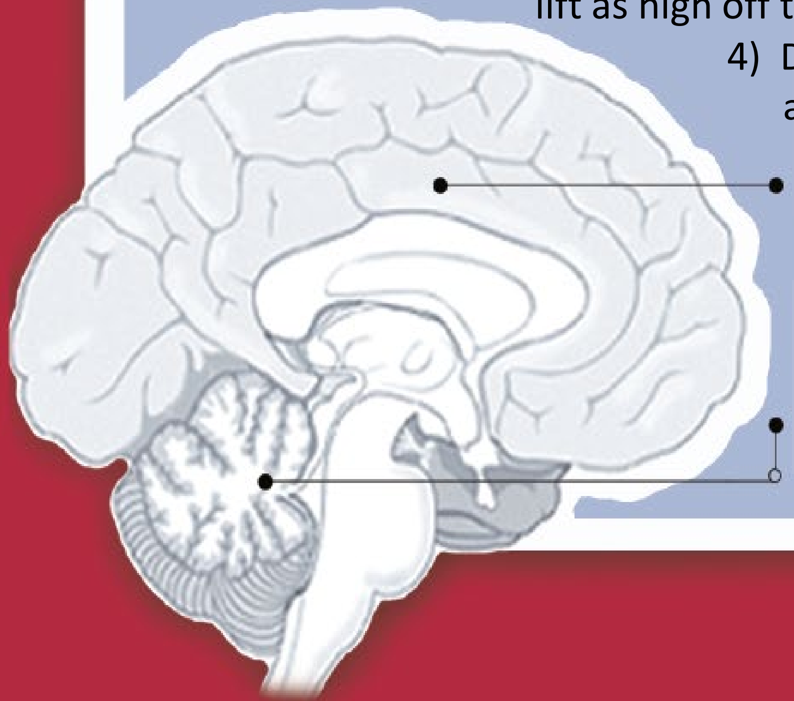

***If there is concern for prior stroke – ask parents how long their child has had these symptoms.**

Instructions

***Quick screen for prior stroke in apparently healthy children in a busy clinic:**

1. Check for pronator drift: have child
hold both hands outstretched, palms up. If one hand drifts down, maybe weaker.
2. Check for smile asymmetry, is one side flattened/won't smile
3. Hand asymmetry /atrophy– is on hand is smaller, atrophied?
4. Tap thumb and finger together (first and second digit opposition)
Watch both hands is one slower and does one hand fatigue faster?
5. Hop up and down on one foot, check both sides. Is one side weaker?
6. Watch the child walk: does one arm swing less than the other side? Do they drag one foot?

If you have concerns, then do full PedNIHSS.

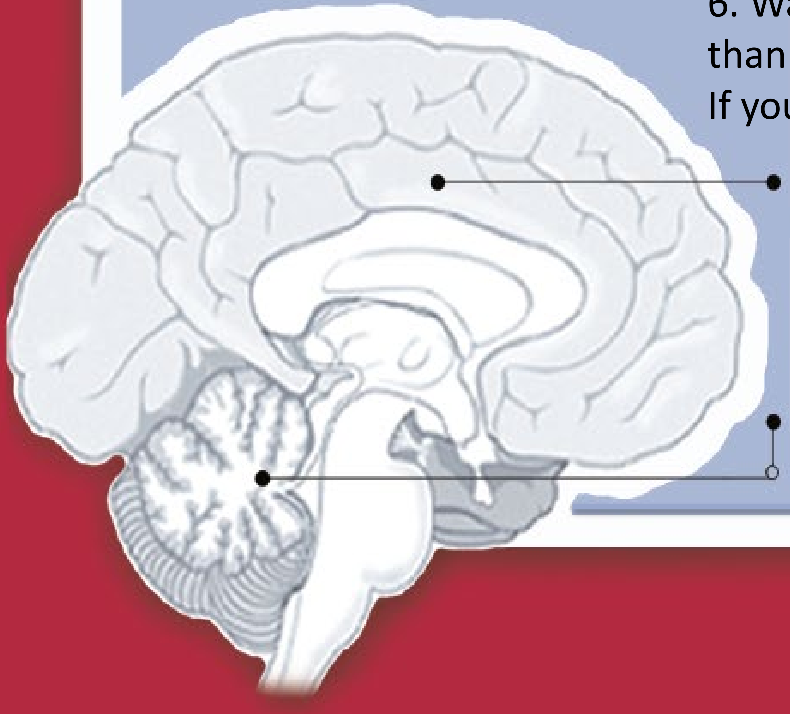

Instructions

For the full PedNIHSS:
Administer stroke scale items in the order listed within this booklet.

Follow directions provided for each exam item.
Record the findings by using the PedNIHSS form provided.

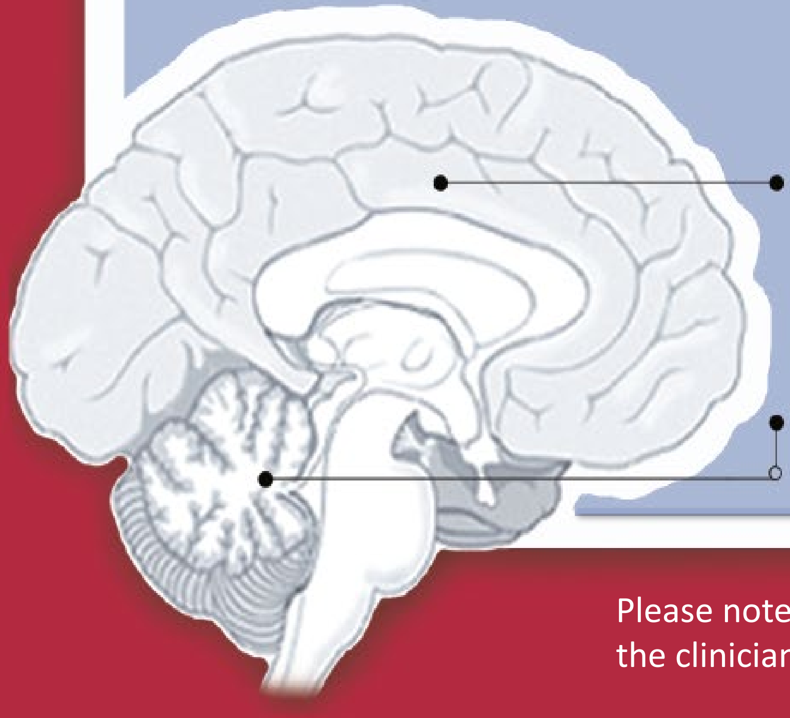

Use these cards for guidance in conducting this exam.

Onset symptoms: Date ___________
(DD/MM/YYYY)

Time ____:_____ (24 hours)

Please note: Scores should reflect what the patient does, not what the clinician thinks the patient can do.

Instructions

Administer stroke scale items in the order listed. Follow directions provided for each exam item. Scores should reflect what the patient does, not what the clinician thinks the patient can do. **MODIFICATIONS FOR CHILDREN: Modifications to testing instructions from the adult version for use in children are shown in bold italic with each item where appropriate. Items with no modifications should be administered and scored with children in the same manner as for adults.**

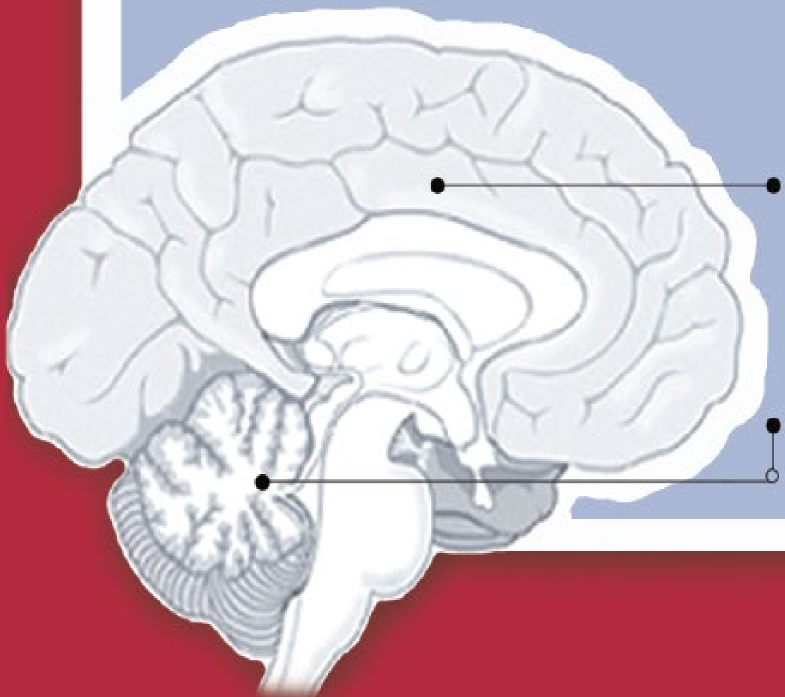

Case ID# ______________________

EXAMINER ______________________

Onset symptoms: Date______________

Time______________________

1a Level of Consciousness

Must choose a response (even if patient has obstacles such as an endotracheal tube, language barrier, or orotracheal trauma/bandages.

Score = 3

- Only if patient makes NO movement to noxious stimuli (other than reflexive posturing)

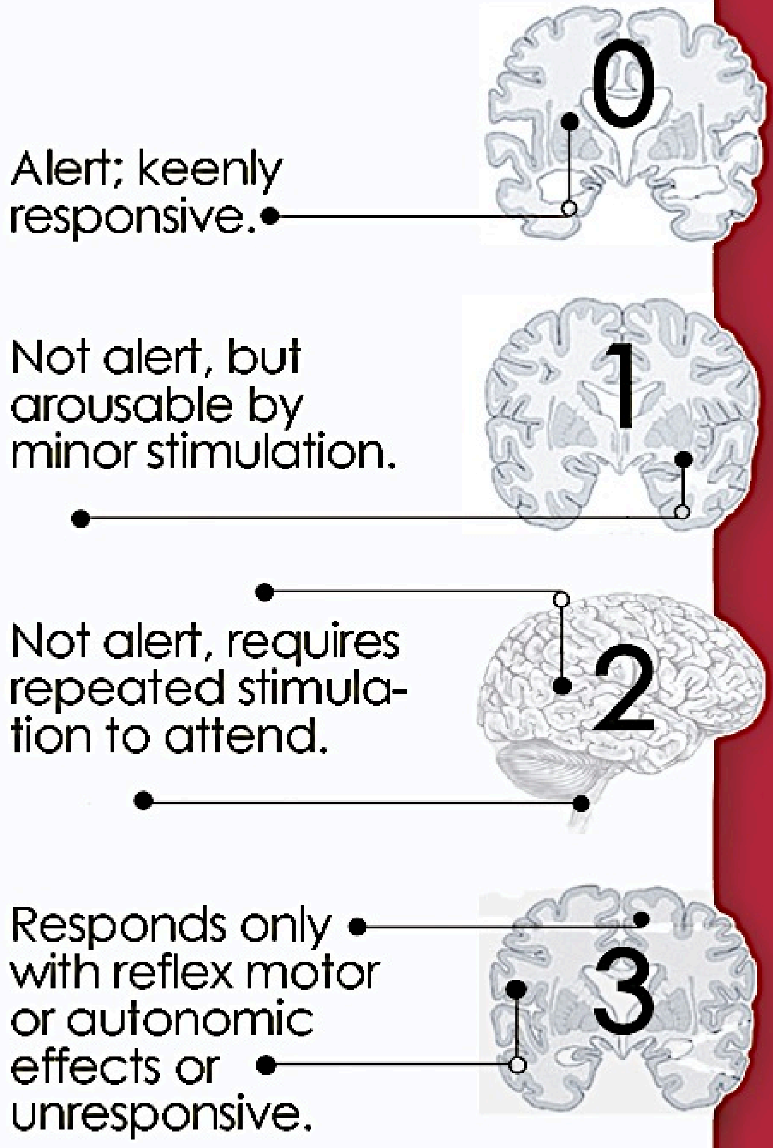

score

1b LOC Questions

A familiar family member **MUST** be present.

- **“How old are you?”**
  - Must speak or show fingers for correct age
- **“Where is ‘mommy’/ ’daddy’?”** **(or familiar family member)**
  - Must correctly point to or gaze toward the direction of this family member

EXCEPTION

Score = 1 Unable to speak due to the following:

- Endotracheal intubation, orotracheal trauma, severe dysarthria (any cause), language barrier, or any problem NOT secondary to aphasia

score

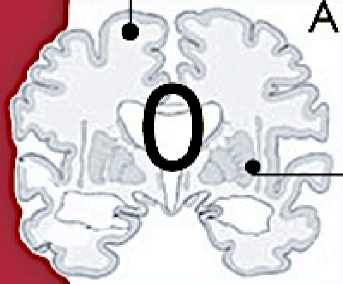

Answers both questions correctly.

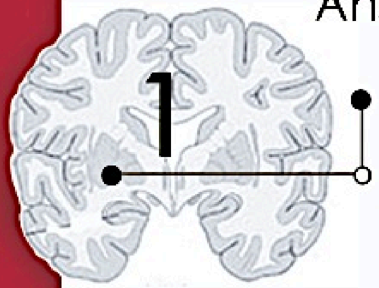

Answers one question correctly.

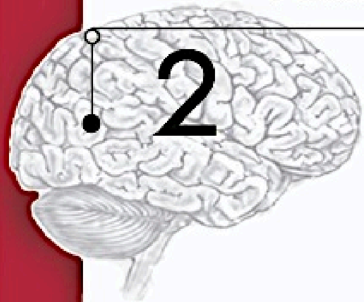

Answers neither question correctly.

1c LOC Commands

<u>ONLY score FIRST ATTEMPTS</u>

- Ask patients to open and close their eyes and do either: "Show me your nose" or "Touch your nose"
  - If cannot use hands, give another one-step command
- Give credit if straightforward attempt made and not completed due to weakness
- If no response to the above, act out task and score as follows: none, one or two commands

Patients with trauma, amputation or other physical hindrance should be given suitable one-step commands.

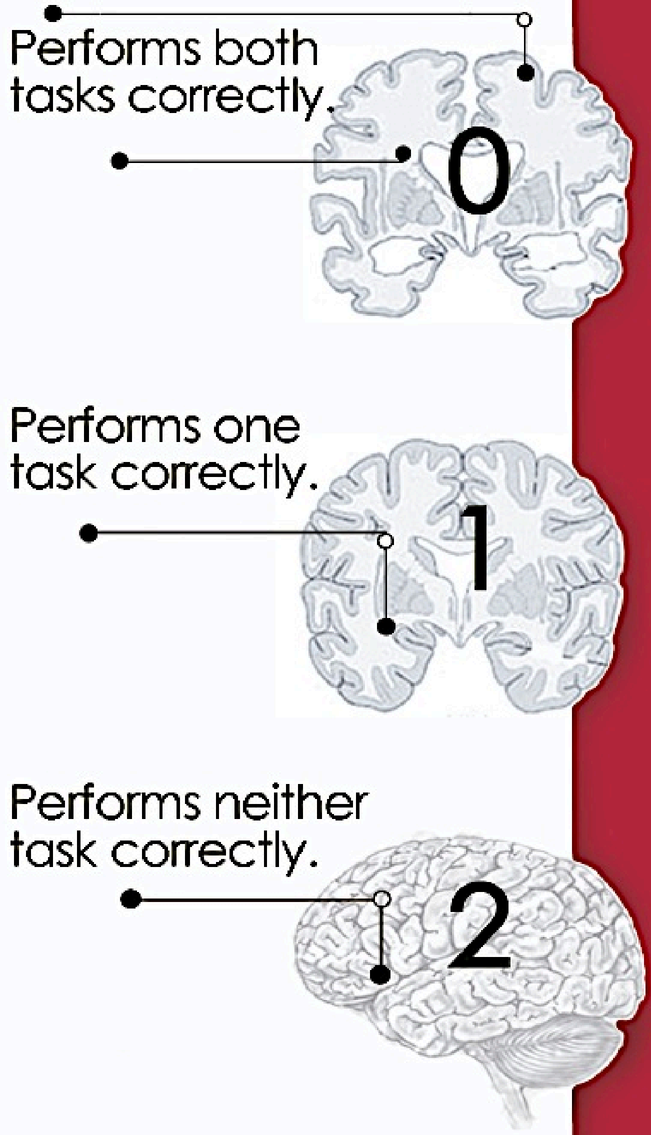

score

Best Gaze

2

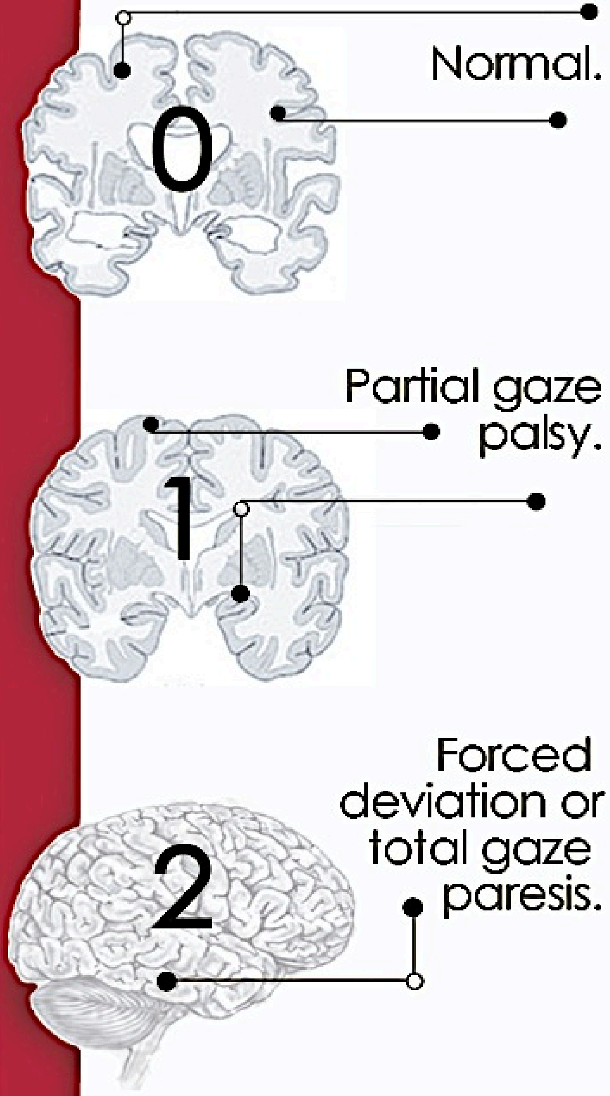

Gaze is testable in ALL aphasic patients

<u>Testing of horizontal eye moments</u>

- Voluntary or reflexive eye movements scored
- Caloric testing <u>NOT</u> scored

<u>Score = 1</u>

- Patient with conjugate deviation overcome by voluntary/reflexive activity
- Isolated peripheral nerve paresis (CN III, IV, VI)

<u>Investigator Chooses Appropriate Score (0 – 2) **ONLY** for:</u>

- Patients with ocular trauma, bandages, preexisting blindness, or other disorder of visual acuity or fields using reflexive movements

score

3 Visual

Upper and lower quadrants tested by confrontation (USE FINGER COUNTING)

If unilateral blindness or enucleation, score remaining eye visual fields.

<u>Normal</u>

- Looks at side of moving fingers appropriately (even if encouraged)

<u>Score = 1</u>

- Clear-cut asymmetry, Quadrantanopia

<u>Score = 2</u>

- Complete hemianopia

<u>Score = 3</u>

- Blind (from any cause)

<u>Double simultaneous stimulation</u> *(results used to answer question 11)*

- Visual extinction present = score as a 1

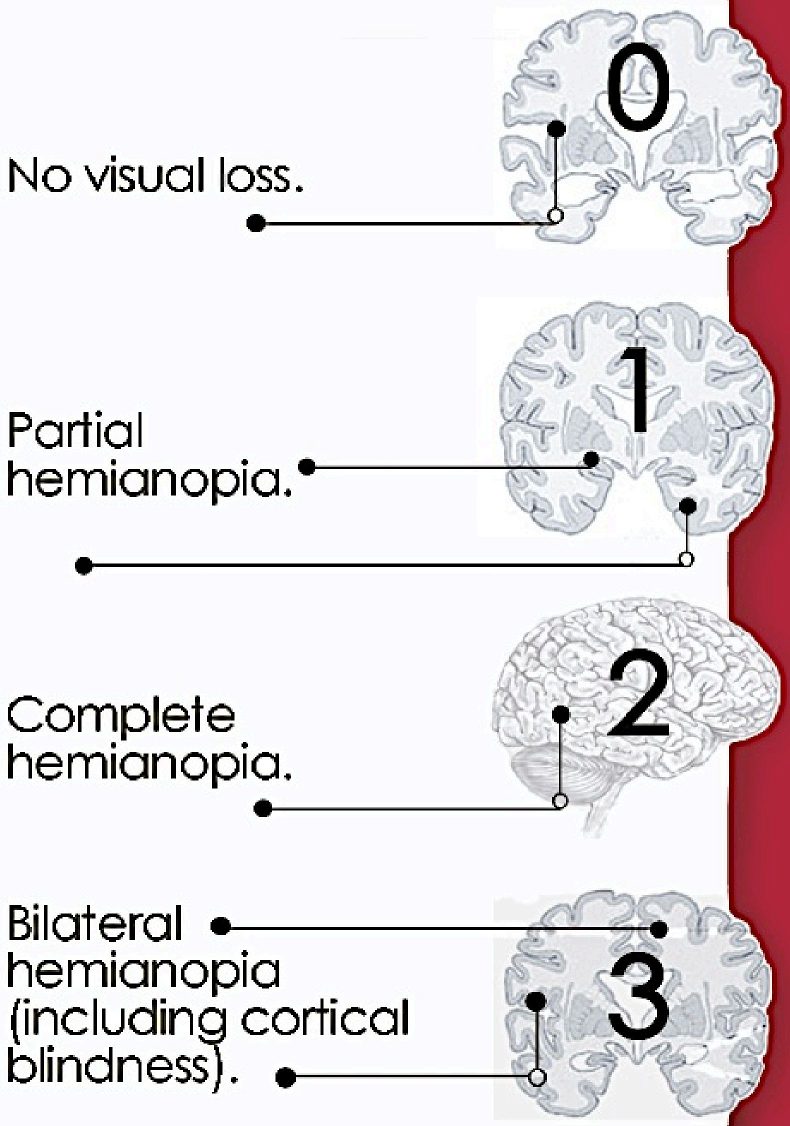

score

Facial Palsy

4

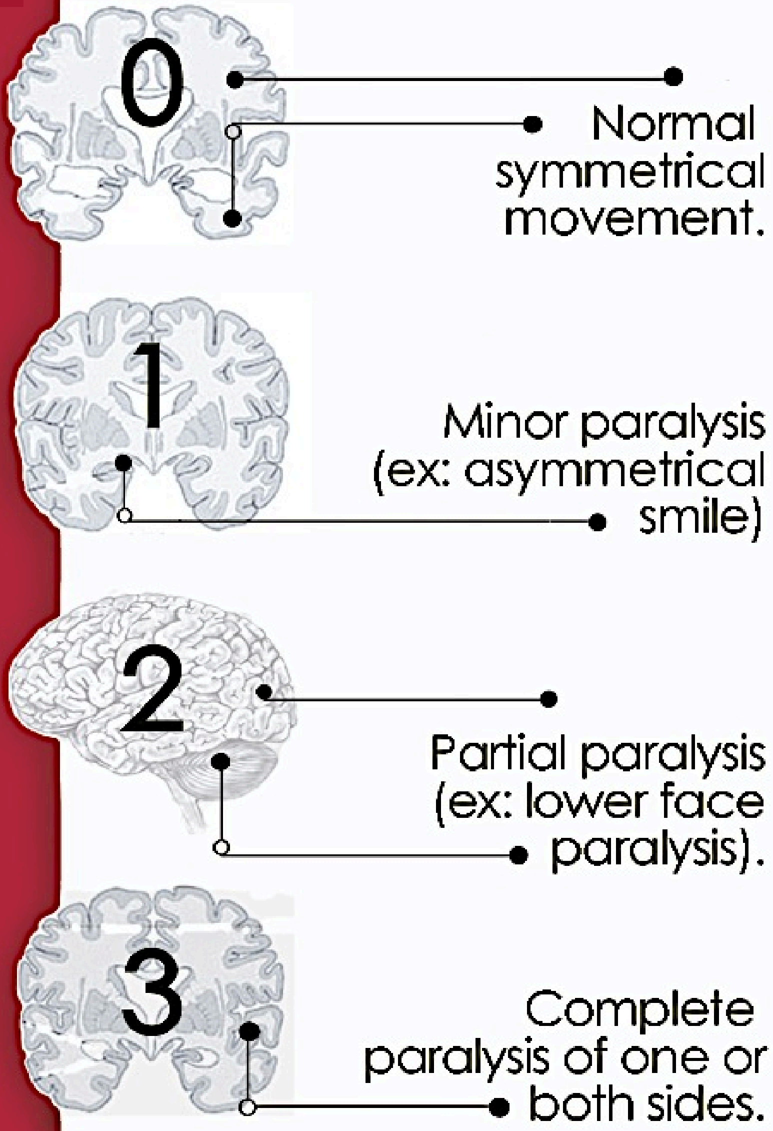

Ask or act out to encourage patient to either:

- Show teeth
- Raise eyebrows

- Score face symmetry in response to noxious stimuli for poorly responsive or non-comprehending patient

Remove facial obstructions below IF possible:

- Facial trauma, bandages, tape or other physical barriers obscuring the patient's face

score

5 Motor Arm and Leg

Place limb in the position below:

- Extend arms (palms down)
  - 90 degrees if sitting
  - 45 degrees if supine

Drift is scored if *arm* falls in *<10 seconds*

Begin in non-paretic arm

- Aphasic patient should be encouraged with voiced urgency (**NO** use of noxious stimuli)

Score = 9 *(clearly write an explanation for score)*

- Amputation or joint fusion at shoulder
- Immobilization by an IV board

Please note: If uncooperative for any reason, grade power in limbs by observing spontaneous or elicited movement according to the above scheme (excluding time limits).

5a. Left Arm
5b. Right Arm

score

No drift, limb holds for full 10 sec.

Drift, limb holds, but drifts down. Does not hit support.

Some effort against gravity, drifts down to support.

No effort against gravity, limb falls.

No Movement.

Amputation or joint fusion.

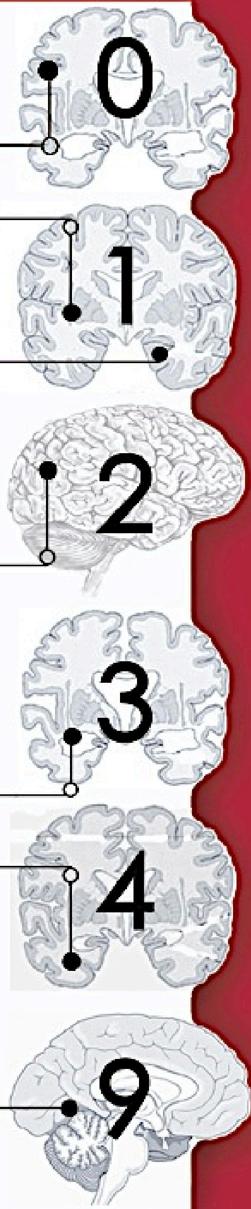

Motor Arm and Leg

6

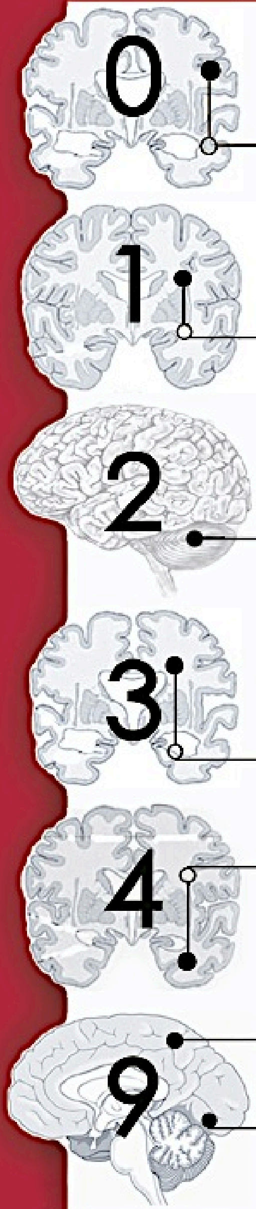

0 — No drift, leg holds 30 degrees for full 5 sec.

1 — Drift, leg falls, but does not hit bed.

2 — Some effort against gravity, falls and hits bed.

3 — No effort against gravity, leg falls immediately.

4 — No Movement.

9 — Amputation or joint fusion.

<u>Place limb in the position below:</u>

- Extend legs 30 degrees (always supine)

Drift is scored if *<u>leg</u>* falls in *<u><5 seconds</u>*

<u>Begin in non-paretic leg</u>

- Aphasic patient should be encouraged with voiced urgency (**<u>NO</u>** use of noxious stimuli)

<u>Score = 9</u> *(clearly write an explanation for score)*

- Amputation or joint fusion at hip
- Immobilization by an IV board

Please note: If uncooperative for any reason, grade power in limbs by observing spontaneous or elicited movement according to the above scheme (excluding time limits).

score

6a. Left Leg
6b. Right Leg

7 Limb Ataxia

To find evidence of unilateral cerebellar lesion

Test with eyes open
Finger-nose-finger and heel-shin tests on both sides

<u>Ataxia</u>

- Scored only if present and not proportionate to weakness
- Absent in patients who cannot understand or are paralyzed

<u>Score = 9</u> (*clearly write an explanation for score)*

- Amputation or joint fusion

Please note: If uncooperative, substitute task with reaching for a toy using the upper extremity, and kicking a toy or examiner's hand.

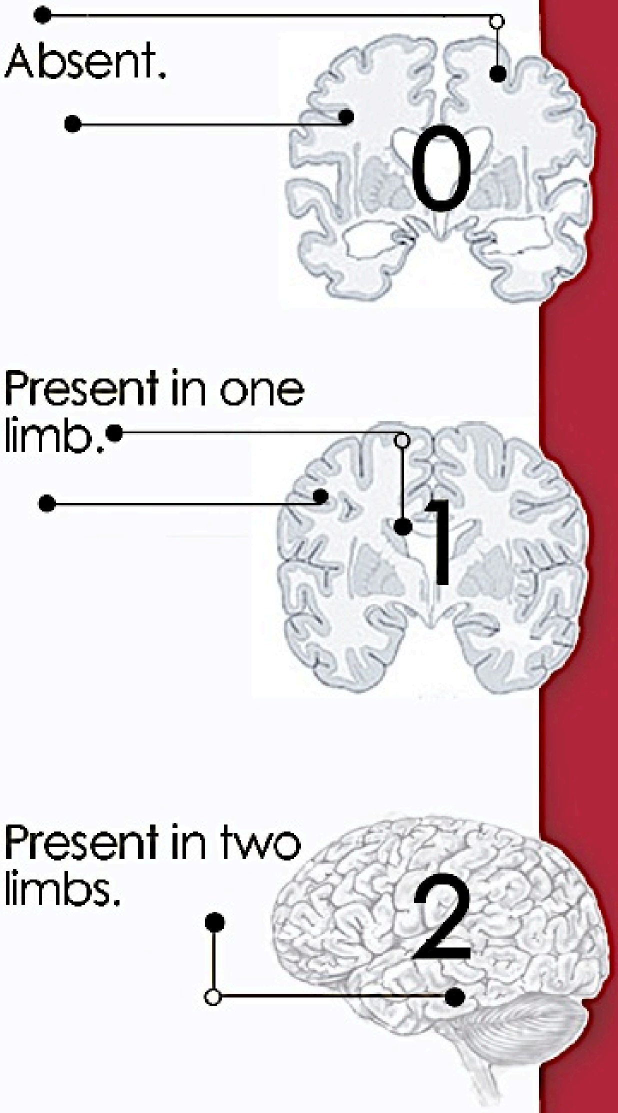

score

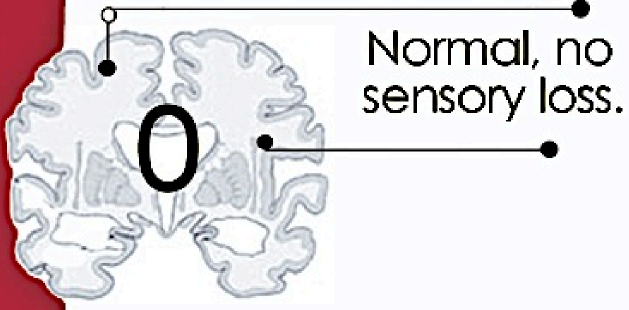

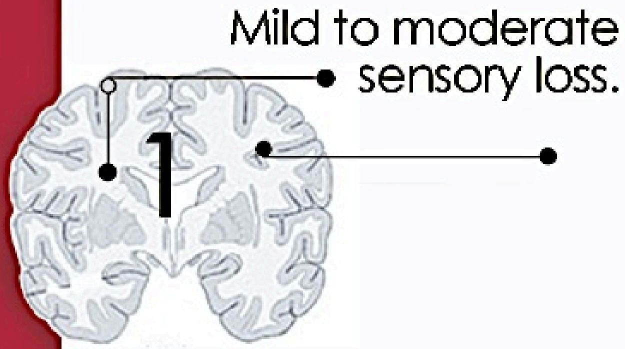

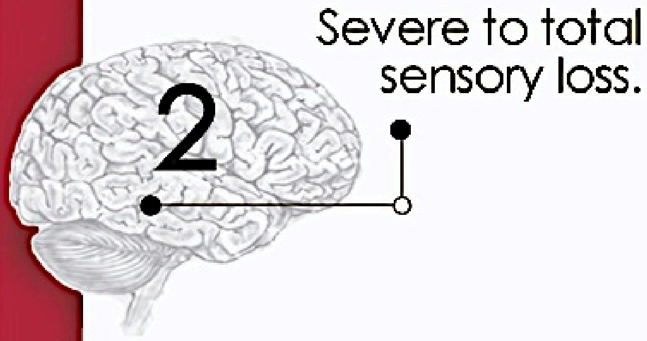

Sensory

8

Pinprick test for sensation or grimace

or

Noxious stimulus in obtunded or aphasic patients for withdrawal

Abnormal sensory loss ONLY if attributed to stroke

- Test as many body areas (arms [not hands], legs, trunk, face)

<u>Score = 0 or 1</u>

- Stuporous and aphasic patients who cannot be assessed

<u>Score = 2</u> *(severe or total loss of sensation)*

- Patient with brainstem stroke with bilateral loss of feeling
- No response
- Quadriplegic
- Coma (question 1a = 3)

Please note: If uncooperative, report gradations of sensory loss by observing for behavioral response to the pin prick and score according to the above scheme as “normal”, “mildly diminished” or “severely diminished”.

score

9 Best Language

Ages 6 and up with normal language development prior to stroke onset:

- Describe the next two slides by naming items and describing pictures
- If patient has vision loss, place objects in his/her hand for identification repeatedly to encourage speaking
- Coma patients (question 1a = 3) will automatically score a 3
- Patients with stupor or limited cooperation should have a score
- chosen but only get a 3 if they are mute and follow no one-step commands

**Comprehension is obtained from these responses and from interaction during the preceding general neurological exam.

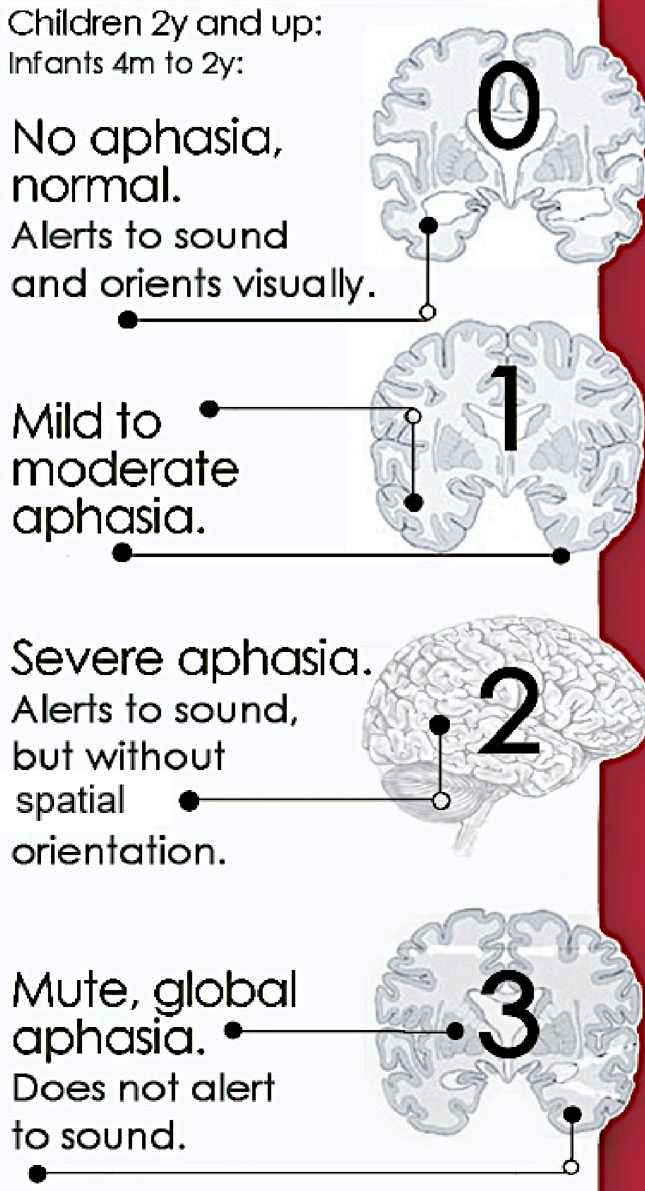

score

Naming Items: Name the pictures

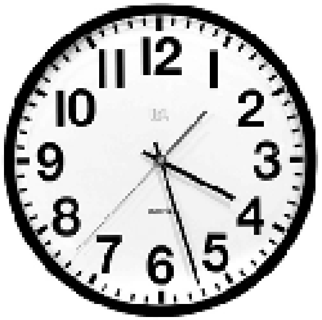

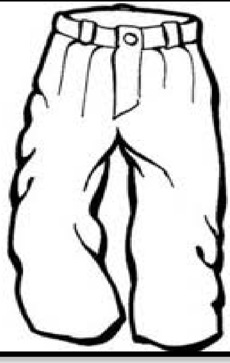

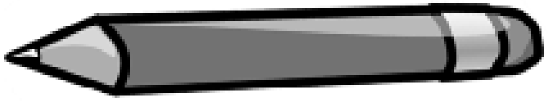

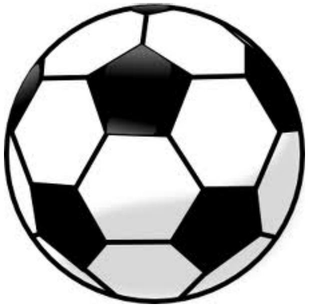

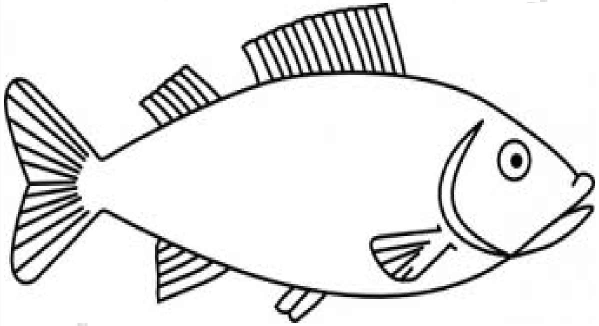

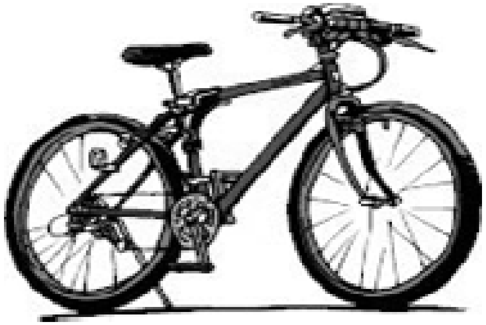

Language Items: Describe the pictures

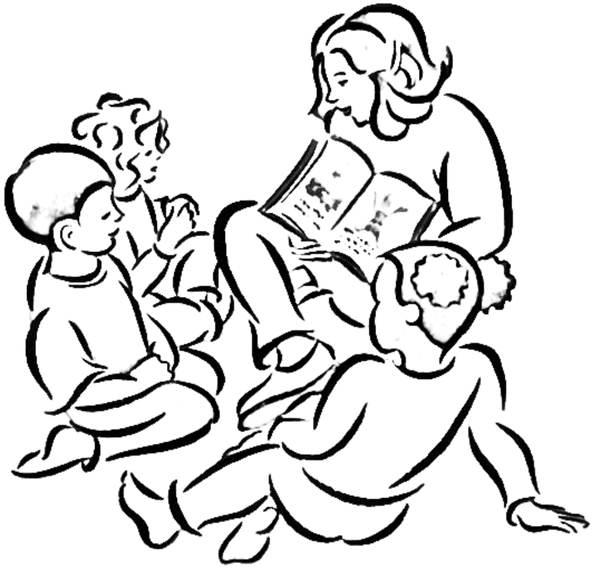

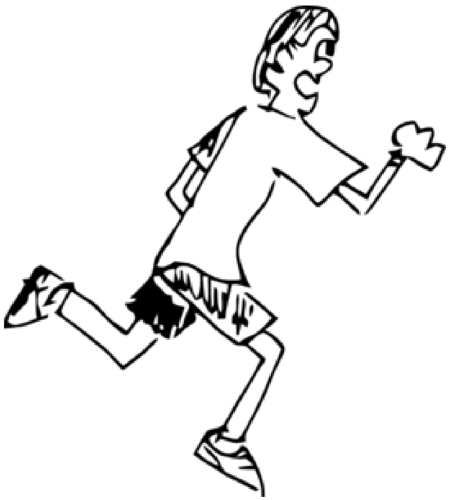

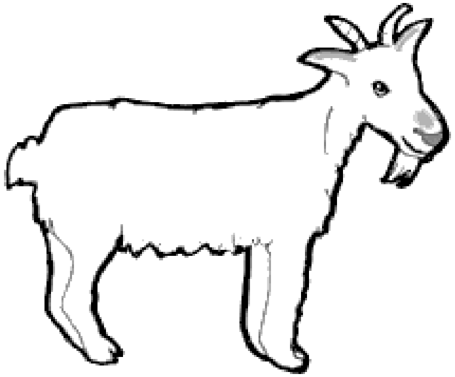

Dysarthria

10

Do not tell the patient that you are assessing speech clarity

Obtain adequate sample of speech by naming and describing pictures

Score = 9
- Only if patient is intubated or has other physical barrier to producing speech
- **MUST** write an explanation of why

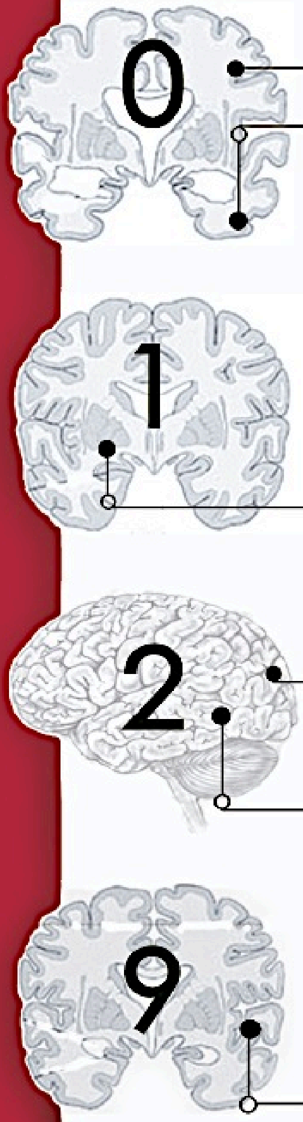

0 Normal.

1 Mild to Moderate (some slurring).

2 Severe (unintelligible).

9 Intubated or other physical barrier.

score

11 Extinction and Inattention

Sufficient information to identify neglect may be obtained during the prior testing

Test attention to light touch on both sides

- Have patient close his/her eyes
- Touch the patient and ask do you feel it on the right/left/both

Test attention to visual stimuli on both sides

Normal

- Severe visual loss preventing visual double simultaneous stimulation and cutaneous stimuli are normal
- Patient has aphasia but appears to attend both sides

Abnormal

- Visual spatial neglect or anosagnosia

This item is **NEVER** untestable since abnormality is only scored if present.

No abnormality.

Inattention or extinction to bilat-eral simultaneous stimulation in one of the sensory modalities.

Profound hemi-inatten-tion or hemi-inattention to more than one modality.

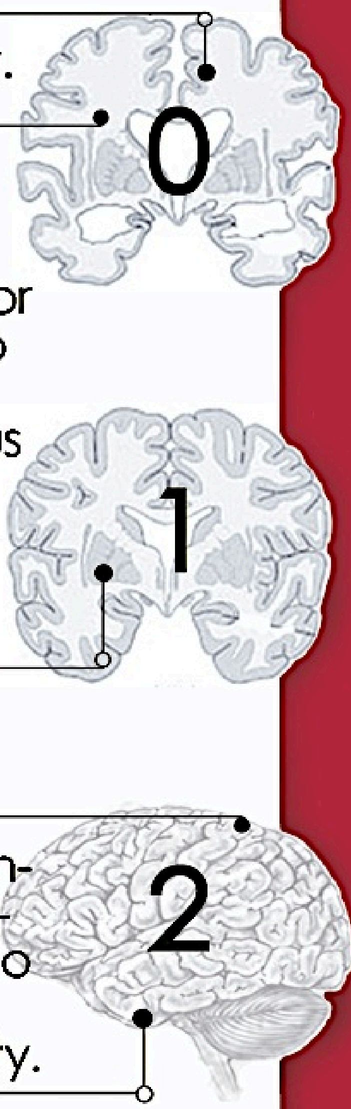

score

If patient not alert due to medications/ sedatives, note on forms.

Scoring of a 9 = not testable and do not include in final total score

Question # and Topic	Possible Scores
1a) LOC	0 1 2 3
1b) LOC: Questions	0 1 2
1c) LOC: Commands	0 1 2
2) Best Gaze	0 1 2
3) Visual	0 1 2 3
4) Facial Palsy	0 1 2 3
5a) Motor: Left Arm	0 1 2 3 4 9
5b) Motor: Right Arm	0 1 2 3 4 9
6a) Motor: Left Leg	0 1 2 3 4 9
6b) Motor: Right Leg	0 1 2 3 4 9
7) Limb Ataxia	0 1 2
8) Sensory	0 1 2
9) Best Language	0 1 2 3
10) Dysarthria	0 1 2 9
11) Extinction and Inattention	0 1 2
**TOTAL**	**Max possible score is 42**

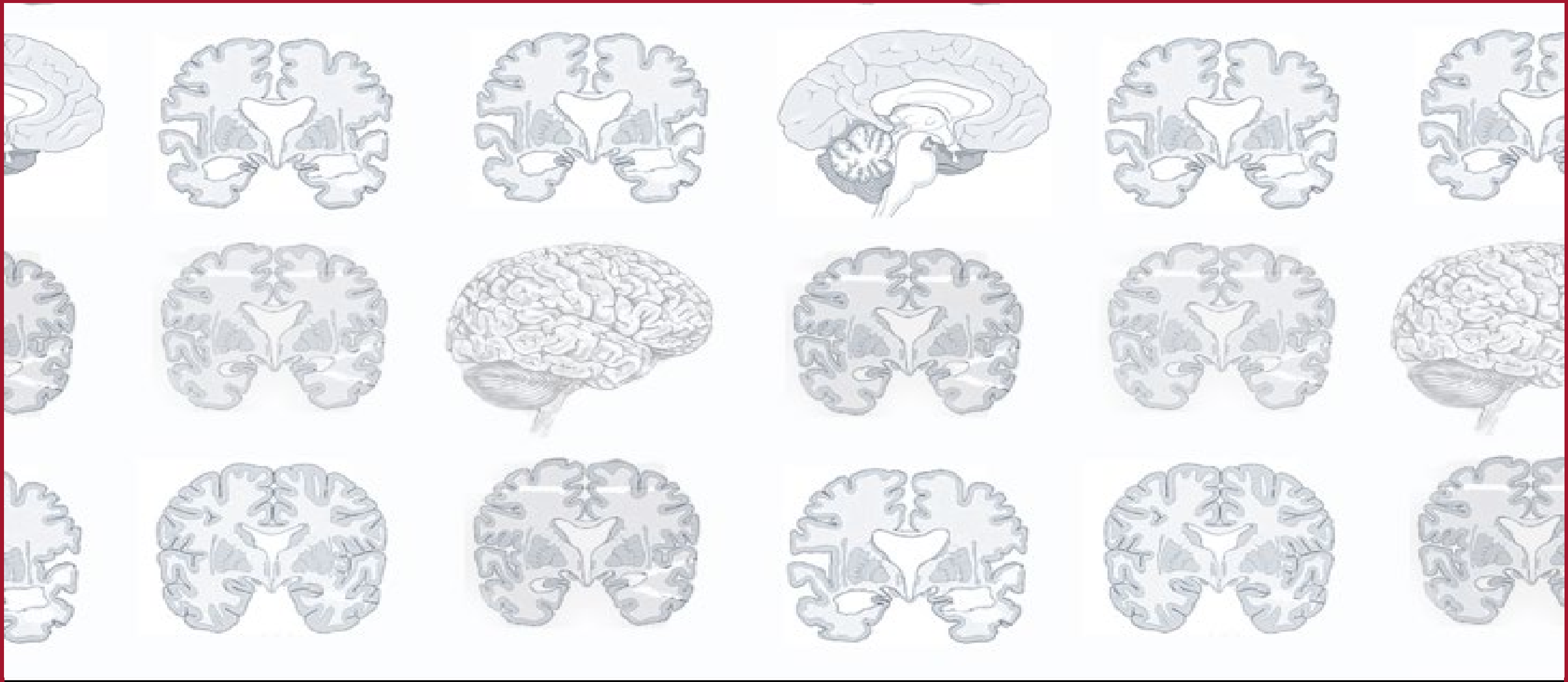

Please fill out the Pediatric NIH Stroke Scale form.

Thank you!!!

Modified for use in Nigeria from the Pediatric NIH Stroke Scale designed and validated by Rebecca Ichord, MD, Director, Pediatric Stroke Program, The Children's Hospital of Philadelphia.

Pediatric NIH Stroke Scale Study

Vanderbilt Meharry Center for Excellence in Sickle Cell Disease
Nashville, Tennessee
