# Supplementary material for: Identifying strokes in Nigerian children with sickle cell disease as part of clinical trials: training curriculum for healthcare professionals in low-income settings
Source: Front Stroke. 2025 Jan 6;3:1444718. doi: 10.3389/fstro.2024.1444718 (PMC12802787; doi:10.3389/fstro.2024.1444718)
Supplement: Supplementary file 2 [file Table_1.DOCX]

**Supplemental Materials: Acute Stroke Care in Nigerian Children with Sickle Cell Disease: Training Curriculum for Healthcare Professionals in Low-Income Settings**

Videos used as part of the Pediatric Stroke Curriculum with links

1. Stroke in Pediatric Sickle Cell Disease Overview <https://www.youtube.com/watch?v=RGRo4IkDFV8>
2. Pediatric Neurology History and Examination -Overview

<https://youtu.be/ldwR0hqNRog>

1. Pediatric Neurology History and Exam Suggesting Stroke

[https://www.youtube.com/watch?v=x9ln2wH41KY](https://nam12.safelinks.protection.outlook.com/?url=https%3A%2F%2Fwww.youtube.com%2Fwatch%3Fv%3Dx9ln2wH41KY&data=05%7C02%7Clori.jordan%40vumc.org%7C4738b5c092dc4a86525208dc8406cabf%7Cef57503014244ed8b83c12c533d879ab%7C0%7C0%7C638530411902137802%7CUnknown%7CTWFpbGZsb3d8eyJWIjoiMC4wLjAwMDAiLCJQIjoiV2luMzIiLCJBTiI6Ik1haWwiLCJXVCI6Mn0%3D%7C0%7C%7C%7C&sdata=hdoQ5GwsJFcPui1CWvJQlcHQoVUZ%2BpichBugPV6uZ2A%3D&reserved=0)

1. Ped NIH Stroke Scale Part 1

<https://www.youtube.com/watch?v=V4MfPVPIywM>

1. Ped NIH Stroke Scale Part 2

<https://www.youtube.com/watch?v=umYuRXwQ3yE>

1. Supportive Care after Ped Stroke in low resource settings

[https://www.youtube.com/watch?v=YkMz1aahtUk](https://nam12.safelinks.protection.outlook.com/?url=https%3A%2F%2Fwww.youtube.com%2Fwatch%3Fv%3DYkMz1aahtUk&data=05%7C02%7Clori.jordan%40vumc.org%7C4738b5c092dc4a86525208dc8406cabf%7Cef57503014244ed8b83c12c533d879ab%7C0%7C0%7C638530411902155448%7CUnknown%7CTWFpbGZsb3d8eyJWIjoiMC4wLjAwMDAiLCJQIjoiV2luMzIiLCJBTiI6Ik1haWwiLCJXVCI6Mn0%3D%7C0%7C%7C%7C&sdata=gdW8idkhb3JK6TbeXf2euNoKCDjudLclTf07IBxLKMw%3D&reserved=0)
